# Effectiveness of an intervention for *Aedes aegypti* control scaled-up under an inter-sectoral approach in a Colombian city hyper-endemic for dengue virus

**DOI:** 10.1371/journal.pone.0230486

**Published:** 2020-04-01

**Authors:** Juliana Quintero, Nicolás Ronderos Pulido, James Logan, Thomas Ant, Jane Bruce, Gabriel Carrasquilla

**Affiliations:** 1 Eje de Salud Poblacional, Fundación Santa Fe de Bogotá, Bogotá, Colombia; 2 Universidad Santo Tomas, Bogotá, Colombia; 3 Department of Disease Control, Faculty of Infectious and Tropical Diseases, London School of Hygiene and Tropical Medicine, London, United Kingdom; Universidade Federal de Lavras, BRAZIL

## Abstract

*Aedes aegypti* transmitted arboviral diseases are of significant importance in Colombia, particularly since the 2014/2015 introduction of chikungunya and Zika in the Americas and the increasing spread of dengue. In response, the Colombian government initiated the scaling-up of a community-based intervention under inter and multi-sector partnerships in two out of four sectors in Girardot, one of the most hyper-endemic dengue cities in the country. Using a quasi-experimental research design a scaled-up community-led *Aedes* control intervention was assessed for its capacity to reduce dengue from January 2010 to August 2017 in Girardot, Colombia. Reported dengue cases, and associated factors were analysed from available data sets from the Colombian disease surveillance systems. We estimated the reduction in dengue cases before and after the intervention using, Propensity Score Matching and an Autoregressive Moving Average model for robustness. In addition, the differences in dengue incidence among scaling-up phases (pre-implementation vs sustainability) and between treatment groups (intervention and control areas) were modelled. Evidence was found in favour of the intervention, although to maximise impact the scaling-up of the intervention should continue until it covers the remaining sectors. It is expected that a greater impact of the intervention can be documented in the next outbreak of dengue in Girardot.

## Introduction

*Aedes aegypti* is the principal vector of dengue, chikungunya, Zika and yellow fever, and is now found in all continents but Antarctica [1]. *Aedes* transmitted diseases account for approximately 23% of the estimated global burden of vector-borne diseases [2] and pose a significant economic cost not only for governments in endemic countries that are responsible for case management and cost of vector control activities, but also for households that have their own costs for treatment and protective measures [3–10].

The emergence and resurgence of *Aedes-*transmitted disease is associated with complex relationships between a variety of ecological, biological, and social factors of urban and peri-urban environments, all of which are particularly challenging for vector control efforts [11–13]. Ecological factors refer to climate (rainfall, humidity, temperature, etc) and the natural and man-made ecological setting (unplanned urbanization). Biological factors relate to the behaviour of the vector, ‬‬‬‬‬‬‬‬‬‬*Ae*. *aegypti*, and transmission dynamics of these diseases (i.e the co-circulation of different serotypes) [14]. Social factors incorporate a series of influences relating to health systems, including the weakening of surveillance systems and vector control programmes[15] and health services [16] and their political context (e.g. health sector reforms, decentralization [17]), public and private services such as sanitation and sewage, garbage collection and water supply. Additionally, "macro-social" events are important, including: demographic growth and urbanization, community and household-based practices, knowledge and attitudes and how these are shaped by large-scale forces such as poverty [18,19], social inequality [20] and community dynamics including human movement [21–23].‬‬‬‬‬‬‬‬‬‬‬‬‬‬‬‬‬‬‬‬‬‬‬‬‬‬‬‬‬‬‬‬‬‬‬‬‬‬‬‬‬‬‬‬‬‬‬‬‬‬‬‬‬‬‬‬‬‬‬‬‬‬‬‬‬‬‬‬‬‬‬‬‬‬‬‬‬‬‬‬‬‬‬‬‬‬‬‬‬‬‬‬‬‬‬‬‬‬‬‬‬‬‬‬‬‬‬‬‬‬‬‬‬‬‬‬‬‬‬‬‬‬‬‬‬‬‬‬‬‬‬‬‬‬‬‬‬‬‬‬‬‬‬‬‬‬‬‬‬‬‬‬‬‬‬‬‬‬‬‬‬‬‬‬‬‬‬‬‬‬‬‬‬‬‬‬‬‬‬‬‬‬‬‬‬‬‬‬‬‬‬‬‬‬‬‬‬‬‬‬‬‬‬‬‬‬‬‬‬‬‬‬‬‬‬‬‬‬‬‬‬‬‬‬‬‬‬‬‬‬‬‬‬‬‬‬‬‬‬‬‬‬‬‬‬‬‬‬‬‬‬‬‬‬‬‬‬‬‬‬‬‬‬‬‬‬‬‬‬‬‬‬‬‬‬‬‬‬

This complexity highlights the need for setting-specific vector control approaches that combine environmental management practices with community mobilization and engagement, intersectoral and multi-stakeholder partnerships, principles of Integrated Vector Management (IVM) [24] as well as other country-specific policies such as the Integrated Management Strategy (IMS) [25].‬ Many community led interventions have been conducted in Asia and results indicate that the interventions reduce vector densities but evidence of impact on dengue transmission is lacking [26].‬‬‬‬‬‬‬‬‬‬‬‬‬‬‬‬‬‬‬‬‬‬‬‬‬‬‬‬‬‬‬‬‬‬‬‬‬‬‬‬‬‬‬‬‬‬‬‬‬‬‬‬‬‬‬‬‬‬‬‬‬‬‬‬‬‬‬‬‬‬‬‬‬‬‬‬‬‬‬‬‬‬‬‬‬‬‬‬‬‬‬‬‬‬‬‬‬‬‬‬‬‬‬‬‬‬‬‬‬‬‬‬‬‬‬‬‬‬‬‬‬‬‬‬‬‬‬‬‬‬‬‬‬‬‬‬‬‬‬‬‬‬‬‬‬‬‬‬‬‬‬‬‬‬‬‬‬‬‬‬‬‬‬‬‬‬‬‬‬‬‬‬‬‬‬‬‬‬‬‬‬‬‬‬‬‬‬‬‬‬‬‬‬‬‬‬‬‬‬‬‬‬‬‬‬‬‬‬‬‬‬‬‬‬‬‬‬‬‬‬‬‬‬‬‬‬‬‬‬‬‬‬‬‬‬‬‬‬‬‬‬‬‬‬‬‬‬‬‬‬‬‬‬‬‬‬‬‬‬‬‬‬‬‬‬‬‬‬‬‬‬‬‬‬‬‬‬‬‬‬‬‬‬‬‬‬‬‬‬‬‬‬‬‬‬‬‬‬‬‬‬‬‬‬‬‬‬‬‬‬‬‬‬‬‬‬‬‬‬‬‬‬‬‬‬‬‬‬‬‬‬‬‬‬‬‬‬‬‬‬‬‬‬‬‬‬‬‬‬‬‬‬‬‬‬‬‬‬‬‬‬‬‬‬‬‬‬‬‬‬‬‬‬‬‬‬‬‬‬‬‬‬‬‬‬‬‬‬‬‬‬‬‬‬‬‬‬‬‬‬

In response to the increasing threat of dengue, the Ecobiosocial/Ecohealth programme was designed by the Special Program for Training and Research in Tropical Diseases (TDR) in partnership with the International Development Research Center from Canada (IDRC) to be implemented in Asia [27] and Latin American countries (Mexico, Ecuador, Colombia, Brazil and Uruguay) over a 4-year period [28]. This initiative carried out a transdisciplinary investigation (Ecohealth approach) of ecological, biological, and social factors of dengue in urban areas, and developed and tested community-based interventions aimed at reducing *Aedes* breeding sites [29].

Specifically, a Cluster Randomized Controlled Trial (CRCT) was conducted in the dengue- hyperendemic Colombian municipality of Girardot during 2012–2014 [30]. The trial was designed to test the efficacy of long-lasting deltamethrin-treated nets (ITN), used as window/door curtains and covers on water containers, in reducing the *Ae*. *aegypti* density as measured through Pupae per Person Index (PPI), a proxy for adult density [30]. The study involved a cluster design comparing ten control and ten intervention areas comprising 100 households each. In control clusters, routine vector control activities (Abate, health education, and occasional public space spraying of an ultra-low volume of Malathion) were conducted. In the intervention clusters, in addition to the routine vector control activities, insecticide-treated curtains were hung over windows and doors and covers were placed over the most *Aedes*-productive water containers. Results demonstrated that PPI in intervention clusters declined by 60% after the intervention with ITN covers.

In light of the results of this trial, and following the recommendations of the 2017 WHO response strategy [2], the Colombian programme decided to extend the intervention in Girardot with the aim of achieving not only broader reduction in vector densities but also impact on dengue transmission. As a key strategy to reach the institutionalisation of the intervention and long-term viability, an intersectoral action approach was implemented among municipal entities from different sectors (health, social development, tourism, academic and education).

Here, we present the impact of an *Aedes*-vector control intervention “Girardot *Aedes*-free” in reducing the number of reported dengue cases in Girardot, Colombia, between 2015 and 2017.

## Methods

### Setting

The study was conducted in Girardot (4°18′02″N 74°48′27″W), Colombia, 134 km from the capital, Bogotá. Girardot is located 289 meters above sea level, has an annual average maximum temperature of 33.3°C, a relative humidity of 66,38%, a mean annual precipitation of 1,220 mm, with two seasons during the year: the dry season (December-April) and the rainy season (May-October). Approximately, 105,085 inhabitants comprise around 23,885 households (97% of which are urban) distributed over 130.32 km^2^. The population triples during the weekends, as its main economic revenue is tourism.

Girardot presents an eco-epidemiological and social niche favourable for sustained transmission of dengue [13,31], chikungunya and Zika. The circulation of multiple dengue serotypes has been reported [32,33]. Between 2005 and 2016, 5,928 dengue cases (residents and non-residents) were reported to the surveillance system from which 5.78% were severe. For this same period, an average of more than 500 dengue cases have been reported annually (range 81–1163). In 2013 1,103 cases were reported, 532 in 2014, and 364 in 2015. The age-groups with higher dengue cases were 5–9 and 10–14 years old (SIVIGILA 2005–2018). With respect to chikungunya the first case in Colombia was identified on September 11^th^ of 2014 and in Girardot on December 2014. By the end of 2015 Girardot, reported 8,905 cases of chikungunya representing an annual incidence of 8,416 per 100,000 inhabitants [34] and by the end of 2016, 1,936 cases of Zika with an overall attack rate of 18.43 per 1000 residents [35].

*Ae*. *aegypti* has been reported as the principal dengue vector in Girardot [31,36] and in other dengue hyperendemic cities of Colombia (Girardot, Armenia, Arauca, Anapoima). The studies report vector productivity associated with storage of water in large and uncovered low level cement containers known as “albercas”, which are estimated to account for more than 70% of pupae production [36–38].

### Study design and data set

An ecological study was conducted to evaluate the impact of an intervention named Girardot *Aedes-free in* reducing notified dengue cases.

Daily dengue surveillance data for the study period January 2010 to December 2017, were obtained from the Communicable Disease Surveillance System of Girardot, Colombia (SIVIGILA) in which patients with dengue are notified according to a standard case definition of dengue [39]. Dengue cases are identified and reported by the health system as either probable dengue, probable severe dengue or, lab confirmed [39].

Anonymized was provided by the health secretary for analysis. In addition, field site access was approved by the city major (Cesar Fabián Villalba) and by the former health secretary (Manuel Díaz) and current health secretary (Erika Ramírez).

### Girardot *Aedes-free* intervention

The intervention focused on four setting levels (household, school, community and institutional) where diverse actors interact and participate with different intervention components together with the control program activities. This intervention was developed, and scaled-up following an Eco health approach [29]. The scaling-up of the intervention occurred in three distinctive phases: 1. Pre-implementation phase (planning and setting-up of activities), 2. Active implementation (action phase) and 3. Sustainability phase (follow-up activities). Table 1, describes the characteristics of the Girardot *Aedes-free* and routine vector control interventions.

**Table 1:** Compared characteristics of Girardot *Aedes*-free intervention and routine dengue control programme in Girardot, Colombia.

Characteristics	*Girardot Aedes*-Free	Routine dengue control program
Actions	*Household level*: Targeted intervention: insecticide-treated covers with aluminium frames or elastic band for *Aedes* productive water containers.	Daily physical inspections of water containers registering presence and absence of immature forms.
		Temephos in tanks. Health education for behavioral change
	*School level*: Community mobilization by students form public schools.	Focal study of severe dengue cases: identification of dengue positive household and surveillance of 40 surrrounding households for spatial fogging, including public spaces.
	*Community level*: Community mobilization by presidents of community boards.	
	*Institutional level*: Intersectoral committee for VBD.	
Human resources	1 field supervisor (environmental engneer). 1 field coordinator (environmental engineer). 4 field technicians (environmental engineers).	11 vector- borne technicians, 1 coordinator, 2 undergraduates as educators.
Household visits	33% of the total of households in each sector (1 and 2) with productive containers.	200 household visits per week, 40 per day in Girardot.
Indexes collected	Immature (presence/absence and pupae per person index) and adult forms.	Presence/absence of immature forms.

**Fig 1:**
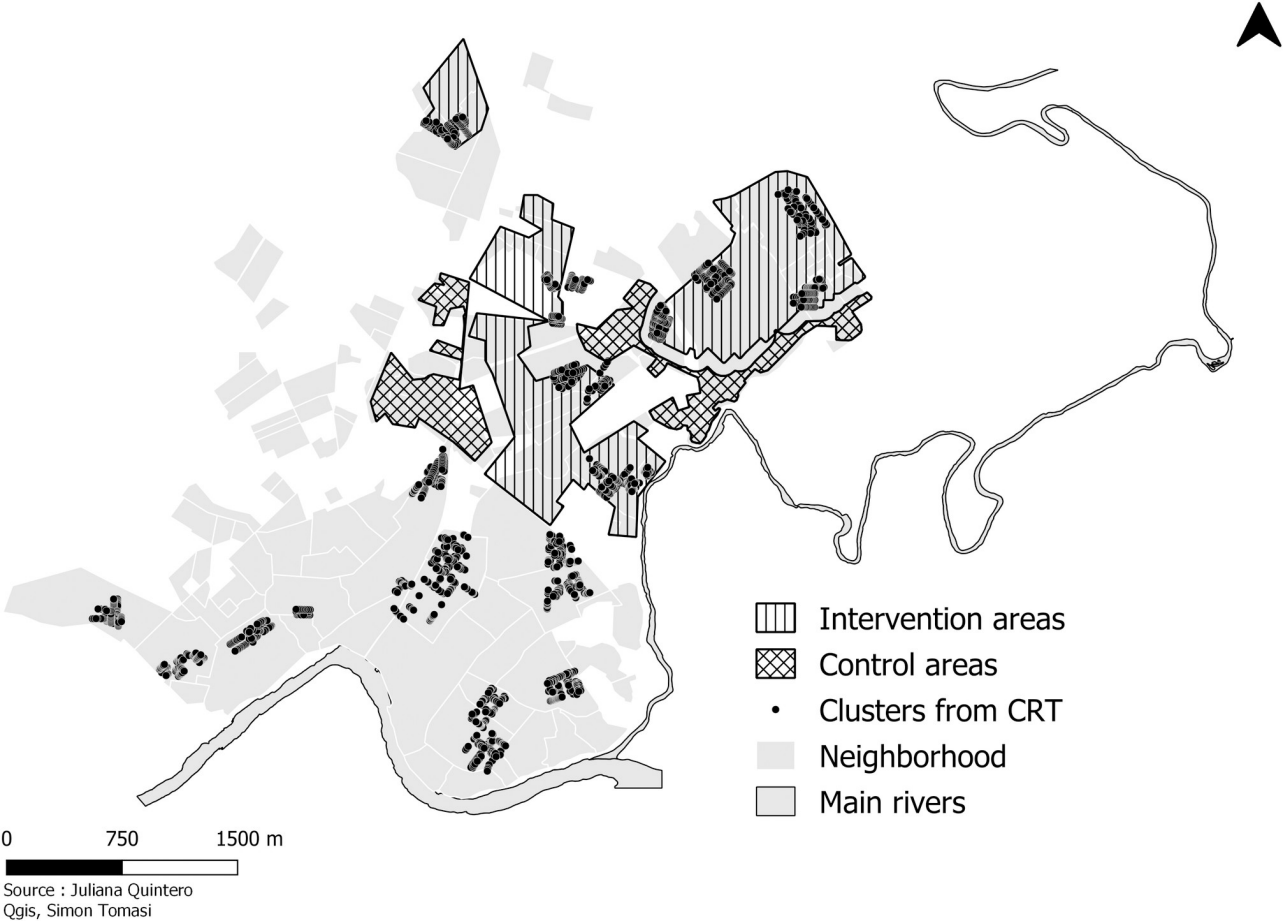
Map of study sectors.

For household level actions, Girardot, was divided into 4 sectors. A sector was defined as an area that included several neighbourhoods with similar ecological and sociodemographic characteristics. Each sector was divided into intervention, buffer (100 meters), and control zones. The active phase began in Sector 1 followed by Sector 2 between December 4^th^ 2015 until February 24^th^ 2017. During the active implementation phase 3,898 insecticide-treated aluminium covers were distributed in 2,935 households (1.32 covers per household) and 1,774 round covers with elastic band in 965 households (1.84 per household). Sectors 1 and 2 represent 2.52 Km^2^ of the total of the urban area of Girardot (130.32 km^2^) (Fig 1).

### Data analysis

The effectiveness of the intervention was assessed using different quasi-experimental research designs. Differences were modelled comparing numbers of reported clinically and lab confirmed dengue cases (primary outcome) among intervention implementation points (before–after) using 1. Propensity Score Matching (PSM) [40,41], (see subsection 2 –below) and an Autoregressive Moving Average (ARMA) model and by modelling differences between numbers of dengue cases adjusted by population size of each sector among scaling-up phases (pre-implementation vs sustainability) and between treatment groups (intervention and control areas) (see subsection 3- below). Difference-in-Differences (Diff–in-Diff) method. All statistical analysis was conducted using Stata software version 15 [42].

1. Propensity Score Matching (PSM)
  The PSM consists of the following steps:

- Estimate the probability that a day would be treated conditional on a set of regressors. The probability is calculated by estimating the coefficients of the model $P(D=1|X)=\Lambda (XB)=\frac{e^{XB}}{1+e^{XB}}$. The coefficients were calculated by maximizing the following likelihood function $L(y_{1},\ldots ,y_{n})=\sum_{i=1}^{n}y_{i}ln(\Lambda (XB))+y_{i}(1-\Lambda (XB))$.
- Check if the score is balanced.
- Match each treated day with one not treated. For this, the following matching algorithms were used:
  - Nearest neighbour matching: Select a pair of control and treated observations that minimize the following expression
    $$Min\|p_{i}-p_{j}\|$$
  - Radius: Select a pair of control and treated observations that fulfil the following expression
    $$\|p_{i}-p_{j}\|<r$$
  - Kernel (Bartlett): Each observation is matched with several observations as:
    $$H(i,j)=\frac{K(\frac{{(p}_{j}-p_{i})}{b})}{\sum_{j}K(\frac{{(p}_{j}-p_{i})}{b})}$$
    Where b is the bandwidth. All alternatives were estimated using common support, a further requirement besides independence.
- Estimate the average impact of treatment on the treated.

#### 2. ARMA

Because the data have a temporal structure, the estimation of an ARMA model (p, q) was performed. The number of cases is the dependent variable.

$$CN_{t}=\frac{e_{t}\beta (L)}{\alpha (L)}$$

Where *L* is the lag operator, i.e *x_t_L^k^* = *x_t−k_*. And the expressions *β*(*L*) and *α*(*L*) are lag polynomials of order *q* and *p* respectively. Using the autocorrelation function, partial autocorrelation and unit root tests, it was determined that the time series has an ARMA structure (3,0,3). To determine the influence of the treatment on the number of cases, a dummy variable was included in the ARMA representation, in the form:

$$CN_{t}=c+\alpha_{1}CN_{t-1}+\alpha_{2}CN_{t-2}+\alpha_{3}CN_{t-3}+e_{t}+\beta_{1}e_{t-1}+\beta_{2}e_{t-2}+\beta_{3}e_{t-3}+\gamma D_{t}$$

After the estimation several diagnostic tests were performed. The estimate is stable, invertible, and its residuals are not autocorrelated.

#### 3. Diff -in- Diff

Differences between numbers of dengue cases (primary outcome) among scaling-up phases (pre-implementation–sustainability) and between treatment groups (sectors 1 and 2) were estimated.

Initially dengue cases were geo-localized using the variable “address” using the SIVIGILA data set (78% of the cases were possible to localize). Then the number of dengue cases was identified in the intervention and control areas per sector using the QGIS software (V 2.18). The QGIS command `Join attributes by location´ was used to create a new vector layer containing information on the number of cases per sectors and intervention areas [43].

A linear regression model was used to estimate the effectiveness of the intervention in the presence of associations between sociodemographic factors reported in the SIVIGILA data set (age, sex, ethnic, health insurance as a proxy of socioeconomic status). A descriptive analysis of baseline and follow-up characteristics was performed for each study group and differences between these characteristics were assessed by bivariate analysis using a test of proportions. Categorical variables were summarized as frequencies and numerical variables as means with standard deviations if normally distributed or as the medians with interquartile ranges (IQRs) if not normally distributed. The regression model used the number of cases as parameter estimates grouped per day. The effect of the intervention was tested as the effect-difference from baseline to follow up between the intervention and control areas. Significance was stated at < 0.05 level and 95% confidence intervals are reported.

### Ethical considerations

International and National Standard Ethical procedures for obtaining protocol approvals as well as Informed consent were followed. The core research proposal entitled “Ecobiosocial approach for the design and implementation of a sustainable strategy for dengue vector in Colombia” was submitted for ethical clearance through the IRB of Fundación Santa Fe de Bogota. Every year the study was updated for ethics approval. The health secretary of Girardot provided and authorized the use of dengue surveillance data which was anonymized prior to access. In addition, ethical approval was obtained by the London School of Hygiene and Tropical Medicine ethics committee under the reference number 14310.

## Results

### Description of notified dengue cases in Giradot, 2010–2017

**Fig 2:**
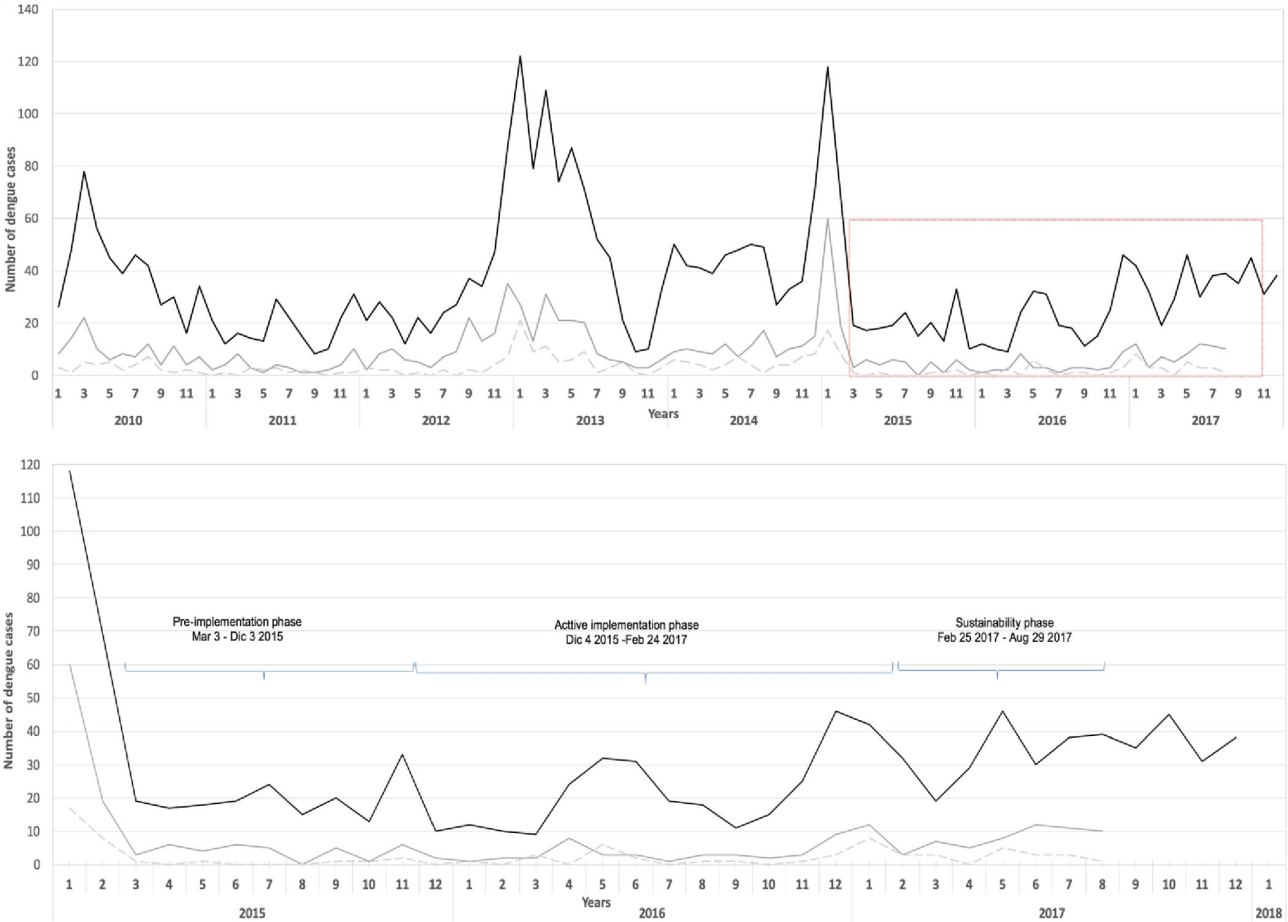
**A.** Number of reported dengue cases in Girardot, Colombia 2010–2017. The solid black line shows the number of dengue cases between 2010 and 2017 in Girardot. The solid gray line shows the number of dengue cases in intervention areas of study sectors 1 and 2. The dashed gray line shows the number of dengue cases in control areas of study sectors 1 and 2. The red square indicates the scaling-up period. **B.** Number of reported dengue cases in control and interventions areas of study sectors. Girardot, Colombia 2010–2017 The solid black line shows the number of dengue cases between 2015 and 2017 in Girardot. The solid gray line shows the number of dengue cases in intervention areas of study sectors 1 and 2. The dashed gray line shows the number of dengue cases in control areas of study sectors 1 and 2.

Between 2010 (1^st^ epidemiological week) and 2017 (33^rd^ epidemiological week), 3,193 suspected dengue cases were reported to the surveillance system of Girardot, of which 99.6% were clinically classified as dengue. During this period a mean of 1.93 dengue cases were reported per day (range 1 to 14) although only 198 (6.2%) were laboratory-confirmed. Fig 2A, shows three outbreaks, over the course of 8 years. During 2010, 487 dengue cases were reported, 708 cases in 2013 and 532 in 2014.

Slightly more dengue cases were reported in men than in women (1,690, 52,9%). The mean age was 21.6 years, and 55.3% (1768) among children younger than 16 years old. A greater number was reported in age-groups 0–5 (587) and 6–10 years old (720).

### Description of notified cases in Giradot, during scaling-up of the intervention

During the period of March 2015 to August 29 2017 (Setup phase: baseline, Active phase: implementation of intervention and Sustainability phase: follow-up) 702 dengue cases were reported in the SIVIGLA of Girardot. Twenty-eight percent of these (n = 194) were from the study sectors, 69 dengue cases were reported during baseline and follow-up in Sector 1, and 52 cases in Sector 2. During baseline 11 cases were reported in the intervention area of Sector 1, compared to 3 cases in the control area.

A similar situation was observed for Sector 2, where 14 dengue cases were reported in the intervention area, compared to 3 cases in the control area. Dengue incidence was generally higher in Sector 1 (526.2 per 100.000 inhabitants) compared to Sector 2 (381.6 per 100.000 inhabitants). For all sectors the incidence was higher in the control area (529.01 per 100.000 inhabitants) than intervention area (371.32 per 100.000 inhabitants). There was an increase in dengue incidence reported in both intervention and control areas from baseline to follow-up. The increase in dengue incidence per 100.000 inhabitants for all sectors was greater in the control areas (an increase of 396.75 cases per 100.000 inhabitants) than in intervention areas (an increase of 267.02 cases per 100.000 inhabitants). In Sector 1, the increase in dengue incidence was higher in control areas (an increase of 483.33 cases per 100.000 inhabitants) than in intervention areas (an increase of 377.43 cases per 100.000 inhabitants). In Sector 2 the incidence in control areas did not change from baseline to follow-up (236.07), but the incidence from baseline to follow-up in the intervention area increased almost two-fold (Table 2).

**Table 2:** Figures of dengue cases in intervention and control areas during baseline and follow-up surveys, Girardot 2015–2017.

Sectors	1				2				All sectors			
Areas	Intervention		Control		Intervention		Control		Intervention		Control	
**Population**	9,538		3,931		14,430		2,118		23,968		6,049	
Number of dengue cases (*)	47 (492.76)		22 (559.65)		42 (291.06)		10 (472.14)		89 (371.32)		32 (529.01)	
**Time of Survey**	**BL**	**FU**	**BL**	**FU**	**BL**	**FU**	**BL**	**FU**	**BL**	**FU**	**BL**	**FU**
**Number of dengue cases per sector**	11	36	3	19	14	28	5	5	25	64	8	24
**Incidence per 100,000 inhabitants**	115.32	377.43	76.31	483.33	97.02	194.04	236.07	236.07	104.30	267.02	132.25	396.75
**Mean age**	25.63		32.63		28.66		23.8		27.06		29.87	
**SD**	24.36		25.26		24.64		26.69		24.40		25.62	
**Sex**												
**F**	22 (46.80)		10 (45.45)		16 (14.28)		5 (50.00)		38 (42.69)		15 (46.87)	
**M**	25 (53.19)		12 (54.54)		26 (61.90)		5 (50.00)		51 (57.30)		17 (53.12)	

BL: baseline, FU: Follow-up, SD: Standard deviations, F: female, M: male.

* Incidence per 100,000 inhabitants.

Table 2, describes the distribution of dengue reported cases in the intervention and control areas during baseline and follow-up surveys only (n = 122).

### Effectiveness

The PSM analysis indicates that the intervention resulted in a decrease of an average of between 0.12 (-0.25,0.01) and 0.26 (-0.42, -0.10) cases of dengue daily (1.82 cases per week or 7.8 cases per month or 95 cases per year) in Girardot (Table 3). By the same means, the time series analysis suggests that the treatment on average decreased the number of dengue cases by 0.27 cases daily (Table 4).

**Table 3:** Average treatment effects estimation using Radius and Kernell matching method.

Matching method	Number of treatments	Numbers of controls	ATT	95% CI	t
Kernel (attk)	215	1414	-0.122	-0.25,0.01	-1.830
Radius (attr)	215	1414	-0.263	-0.42, -0.10	-3.170

Number of observations = 1629 Replications = 2500, ATT: Average treatment effect on the Treated group, CI: Confidence Interval.

**Table 4:** Number of dengue cases after intervention estimated by Arma model.

Variables	Coefficient	95% CI	p-value
Constant	1.86	1.50, 2.22	<0.0001
Intervention	-0.27	-0.95, 0.41	0.436
ARMA parameters			
AR (1)	1.68	1.25, 2.10	<0.0001
AR (2)	-0.68	-1.10, -0.261	0.002
MA (1)	-1.54	-1.97, -1.11	<0.0001
MA (2)	0.54	0.19,0.89	0.002
MA (3)	0.01	-0.06,0.09	0.721
Sigma	1.23	1.20,1.26	<0.0001

AR: Auto Regressive, MA: Moving Average, CI: Confidence Interval.

The Diff-in-Diff estimator reports an increase of 0.065 dengue cases daily (0.455 per week, 1.95 per month) (Table 5), but when calculating the differences in incidences rates and rate ratios during sustainability (follow-up phase) among intervention and control areas of both sectors (see Table 2), an incidence rate difference of– 0.0129 (95% CI -0.00179- -0.00078) and an incidence rate ratio of 0.674 (95% CI 0.577–0.786) are observed.

**Table 5:** Difference-in-Difference estimation results from sectors 1 and 2, Girardot.

Outcome variable	Dengue cases	Standard Error	p-value	95% CI
Baseline				
Control	0.929			
Treated	0.989			
Diff (T-C)	0.060	0.069	0.387	-0.0763538 0.1961956
Follow-up				
Control	0.950			
Treated	1.075			
Diff (T-C)	0.125	0.086	0.151	
Diff-in-Diff	0.065	0.1120	0.557	-0.152547 0.2818859

R-square: 0.06, Means and Standard Errors are estimated by linear regression, Number of observations in the Diff-in-Diff: 173. Adjusted by age, sex, season and health insurance.

## Discussion

The principal goal of any dengue intervention is to reduce disease incidence and preferably transmission by reducing human exposure. *Ae*. *aegypti* control remains the primary tool available to achieve the latter goal. Several systematic reviews published in the last decade [26,44–47] have reported the impact of dengue vector control. They concluded that the most effective are community-based interventions that combine community and social mobilization, participation with local government control services, joint collaboration with local services, with environmental management or clean-up campaigns, water covers and window screens using insecticide-treated nets, and use of larvicides.

These reviews indicated that the effect of a dengue vector control interventions is principally measured using entomological parameters (indicators of vector infestation) however these indicators do not always accurately reflect dengue transmission [48,49]. The studies that include epidemiological risk indicators to determine the effect of a dengue vector control intervention mainly use interrupted time series, propensity score matching and classic, spatial, and Bayesian statistical analysis, [50–52]. These are preferably selected as costs, resource demands, and contamination effects are factors that impede the feasibility of conducting alternative experimental designs.

Our study used a quasi-experimental design to assess the impact of a dengue vector control intervention in Girardot, Colombia, developed with local stakeholders and implemented following an ecohealth approach [26].

When analyzing the series of dengue cases, it is observed that outbreaks occur every three years and importantly, the number of cases per season differed, more cases were reported in the dry seasons than in the rainy seasons. Although the dry season includes 7 months (December–February and June–September), and the rainy season includes only 5 (March–May and October–November), the former still has a higher mean number of cases per month. High temperatures, relative humidity, precipitation are well known factors that related to dengue transmission [53,54]. These factors facilitate *Ae*. *aegypti*, population growth, but in Girardot the increase in tourism (presence of susceptible populations, higher population density) during the dry seasons is also an important factor that favors virus transmission and can have an effect on the increase of dengue cases reported during this season.

Despite low dengue transmission reported during the intervention phases the results indicate that the areas covered with the intervention reported a reduced dengue incidence over the 6 to 12 months compared to control areas, although in both areas the incidence increased. The difference in dengue incidences seen per sector after the implementation phase may be due to the reduced use of container covers over time. Follow-up of sector 1 was performed 12 months after intervention implementation compared to sector 2, where follow-up was performed after 6 months. A variety of studies argue that the use of an intervention tends to decrease over time [55–57]. As with any vector control measure, a consistent level of compliance is needed by household members to gain sustainability of the intervention [58,59]. There is a need for identifying factors capable of achieving permanent changes in human behavior. Moreover, the percentage of productive container coverage per sector did not reach 100% (Sector 1, 39.54% of coverage and Sector 2, 50.39% of coverage). High coverage is needed for the intervention to have a broader impact [55–57]. The main reason for the limited coverage was the inaccessibility of participant houses, even after three visits. It is important to point out that Girardot is a touristic site and many of the houses are the “second residences” of inhabitants of other cities (primarily Bogotá) for recreational purposes [37].

More than half of the study population comprised schoolchildren who attend school during the daytime. Schoolchildren participated in mobilization activities but not breeding-site interventions nor were screens for classrooms implemented in schools. Another important age group are young men and women who spend significant proportions of time in places of work and in commercials sites, neither of which were included as interventions sites. A growing body of evidence [22,60–62]has shown that human movement is an important consideration when analysing the effectiveness of vector interventions and understanding dengue epidemiology. Previous studies have shown that transmission of dengue virus appears to be largely driven by infections centered in and around the home, with the majority of cases related to one another occurring in people who live less than 200 meters apart, supporting a role for targeted vector control around the residences of detected cases [63].

Furthermore, it is important to consider other locations where individuals tend to gather and spend significant amounts of time, as they may play an important role in the virus cycle. Sites such as schools pose a risk of transmission as there may be abundant breeding containers for *Aedes* vectors and will contain an aggregation of students during the daytime [64–66].

Defining effectiveness is one of the elements for scaling-up an intervention into public health policies. However, other criteria are equally important, including acceptability, reach, adoption, ease of delivery, alignment with local policies and cost [67–69]. Further analysis of the fidelity [70–72] of the intervention and cost effectiveness are being conducted to have a broader picture for decision making.

### Limitations

Using secondary quantitative information of notified dengue cases from SIVIGILA possess several challenges, which has been evident in studies in other countries such as Colombia [32,73–75]. The surveillance system only captures symptomatic patients who sought treatment at health care services, and are registered with a residential address that is not necessary the location of dengue transmission. In addition, no specific serotype information is reported.

Another limitation is the available spatial information (road network, neighbourhoods and blocks) and the address and neighbourhood fields in the SIVIGILA database required for identifying dengue cases. The address and neighbourhood fields are not standardized and an important work of filtering information was needed to decrease the error when localizing each dengue case by sectors and intervention and control areas; but, seventy eight percent of dengue reported cases were able to geo-locate, underestimating the true incidence.

In addition, during intervention dengue incidence decreased during this period following the trend of dengue peaks in Girardot and elsewhere every three years. It would be expected that at higher incidence of dengue the impact of intervention may have been higher than the one reported in our follow up. The decrease of dengue cases during the scaling-up phases can be a long-term result from previous interventions carried out in Girardot since 2012 combined with enhanced vector control actions implemented by the local health authorities due to the re-emergence of chikungunya and Zika viruses.

## Conclusion

The aim of dengue vector control is to maintain *Ae*. *aegypti* populations below or close to minimal transmission thresholds, slow the force of dengue-virus transmission, and reduce sequential infections with different serotypes. Here an intervention was evaluated for its capacity to reduce notified dengue cases by targeting the most productive dengue vector containers. The results indicate a reduction in dengue incidence compared to matched controls sites, although this is probably an underestimated of the true potential of the intervention. Greater coverage, reaching other sectors and other high-risk transmission areas (Public spaces such as school and commercial sites), and improved surveillance system are required for maximising the effect of the intervention.

## Supporting information

S1 Data Girardot dengue cases 2010 2017. (XLSX)

S2 Data Data base for Diff in diff estimation. (XLSX)
